# Deep Learning-based Assessment of Internal Carotid Artery Anatomy to Predict Difficult Intracranial Access in Endovascular Recanalization of Acute Ischemic Stroke

**DOI:** 10.1007/s00062-023-01276-0

**Published:** 2023-03-16

**Authors:** Gregor Nageler, Ingmar Gergel, Markus Fangerau, Michael Breckwoldt, Fatih Seker, Martin Bendszus, Markus Möhlenbruch, Ulf Neuberger

**Affiliations:** 1 Dept. of Neuroradiology, Heidelberg University Hospital, Im Neuenheimer Feld 400, 69120 Heidelberg, Germany; 2 mbits imaging GmbH, Heidelberg, Germany

**Keywords:** Machine learning, Mechanical thrombectomy, Tortuosity, nnUNet, Convolutional neural network (CNN)

## Abstract

**Background:**

Endovascular thrombectomy (EVT) duration is an important predictor for neurological outcome. Recently it was shown that an angle of ≤ 90° of the internal carotid artery (ICA) is predictive for longer EVT duration. As manual angle measurement is not trivial and time-consuming, deep learning (DL) could help identifying difficult EVT cases in advance.

**Methods:**

We included 379 CT angiographies (CTA) of patients who underwent EVT between January 2016 and December 2020. Manual segmentation of 121 CTAs was performed for the aortic arch, common carotid artery (CCA) and ICA. These were used to train a nnUNet. The remaining 258 CTAs were segmented using the trained nnUNet with manual verification afterwards. Angles of left and right ICAs were measured resulting in two classes: acute angle ≤ 90° and > 90°. The segmentations together with angle measurements were used to train a convolutional neural network (CNN) determining the ICA angle. The performance was evaluated using Dice scores. The classification was evaluated using AUC and accuracy. Associations of ICA angle and procedural times was explored using median and Whitney‑U test.

**Results:**

Median EVT duration for cases with ICA angle > 90° was 48 min and with ≤ 90° was 64 min (*p* = 0.001). Segmentation evaluation showed Dice scores of 0.94 for the aorta and 0.86 for CCA/ICA, respectively. Evaluation of ICA angle determination resulted in an AUC of 0.92 and accuracy of 0.85.

**Conclusion:**

The association between ICA angle and EVT duration could be verified and a DL-based method for semi-automatic assessment with the potential for full automation was developed. More anatomical features of interest could be examined in a similar fashion.

**Supplementary Information:**

The online version of this article (10.1007/s00062-023-01276-0) contains supplementary material, which is available to authorized users.

## Introduction

Endovascular thrombectomy (EVT) is an established component in treating acute ischemic stroke (AIS) patients with large vessel occlusions (LVO) [1]. Recently it was demonstrated that EVT duration is among the leading predictors for the long-term neurological outcome [2, 3].

Prediction of and clinical outcome for EVT is an active field of research showing promising results [4–10]. Specifically, multiple studies have investigated the influence of the vascular access to the occlusion site. Features that were investigated included the configuration of the aortic arch as well as of the common carotid artery (CCA) and intracranial carotid artery (ICA) [11–16]. One study proposed a semi-automatic approach to assess vascular tortuosity but did not elaborate on how much manual work must be invested in obtaining the proposed tortuosity index [17]. Recently, a study with a larger patient collective of *n* = 828 was able to show that an acute inner angle of ≤ 90° in the ICA is associated with a longer EVT duration [18].

Artificial intelligence in the diagnosis and management of AIS is a growing field [19–21]. It was previously demonstrated that methods using deep learning (DL) are viable for the automatic segmentation of neck vasculature [22, 23], LVO detection [24] and outcome prediction based on the intracranial vasculature [25].

This work aims to develop a DL-based combined segmentation and classification approach to assess for critical vascular anatomy regarding EVT, specifically an acute ICA angle ≤ 90°.

## Methods

### Patient Selection

Consecutive patients who were treated with EVT for LVO of the M1 segment of the middle cerebral artery between 2016 and 2020 (*n* = 447) in a tertiary care center were included. To emphasize the effects of difficulties mainly in probing the ICA, distal occlusions as well as carotid‑T occlusions were excluded, as in those cases probing might be complicated not mainly due to the ICA anatomy, but because of missing contrast due to pseudo-occlusion or due to sharp angles of M2 branches and superimposition of overlaying vessels.

**Fig. 1:**
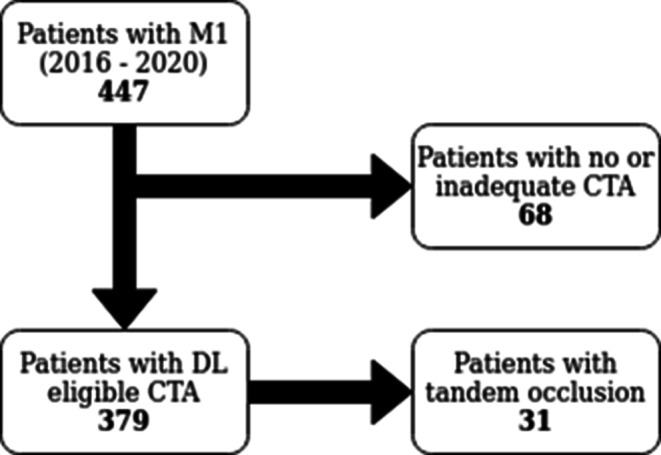
Inclusion flowchart detailing the process of patient selection

Of the 447 patients included initially, 68 had to be excluded due to unavailability of CTA. Among the remaining 379 patients, 31 patients had a tandem occlusion (concomitant occlusion of the proximal or cervical ICA). Data of these patients were used for the development of the DL models as the vascular anatomy was still valid for learning purposes; however, in the analysis of procedural times in relation to the vessel angle, patients with tandem occlusion or inadequate contrast filling of the ICA, either due to higher grade proximal stenoses that were not treated in the acute setting, or due to late contrast phases, were not included. Figure 1 shows an overview of the patient selection.

The CTA data were exported from the picture archiving and communicating system (PACS) to a local machine for further analysis. Table 1 summarizes the distribution of the Y‑axis voxel spacings which were in the majority 0.5 mm in the Y‑axis voxel spacing (*n* = 221). CTA data of a lower Y‑axis resolution was also considered to obtain networks that perform on heterogeneous input regarding the imaging quality.

**Table 1:** Distribution of Y‑axis voxel spacings

Z‑axis voxel spacing	< 1 mm	≥ 1 mm, < 2 mm	≥ 2 mm
*N*	322	30	27

For the development of the DL models a train/test split was performed, with 15% of the cases being allocated to the test set resulting in 321 train and 58 test cases.

### Vessel Tree Segmentation

In order to achieve information reduction and to specifically select anatomical structures for classification, a DL-based vessel segmentation was developed, 121 CTAs were segmented manually (3D Slicer with a 3-dimensional sphere brush [26]) and 3 distinct labels were assigned to the different anatomic sectors during manual segmentation. Label 1 was given to the aorta, label 2 to the left CCA + ICA, and label 3 to the right CCA + ICA.

Of the labeled cases, 100 belonged to the training set and 21 to the test set. On the training set, a nnUNet [27] was trained. The training mode used was 3d_lowres with the nnUNetTrainerV2_noMirroring trainer class. The training ran for 1000 epochs. The resulting model was evaluated on the test set using Dice score, precision, and recall.

In order to make use of the trained nnUNet for the segmentation of the remaining 258 CTAs, a hybrid segmentation approach was conceived. This approach consisted of 2 steps:

1. Prediction of a segmentation by the trained nnUNet.
2. Manual verification of the predicted segmentation.

Manual verification was performed by overlaying the predicted segmentation onto the respective CTA. The integrity of the segmentations was checked in this fashion, and corrections were performed if necessary.

The time the trained nnUNet took to predict the 258 cases was registered. The mean time per segmentation for the manual correction was obtained by counting the number of segmentations finished during an exemplary 8 h working day. Additionally, the median time per segmentation was acquired in a subset of cases (*n* = 50).

### ICA Angle Measurement

In order to obtain a training set as large as possible for the DL development, both ICAs of each patient were used. Left and right ICAs were assessed regarding the most acute angle in their course. The assessment was performed using the angle measurement tool in 3D Slicer. The measurement was performed using the angle measurement tool in 3D Slicer which enables angle measurement on the surface of segmentations. The standard CTA reconstruction planes often do not match the plane of the vessel course. By setting the angle measurement points on the vessel surface a plane is projected onto it. This plane is a close approximation of the true plane of the course of the vessel. It therefore allows for more precise angle measurement than on standard CTA reconstruction planes.

**Fig. 2:**
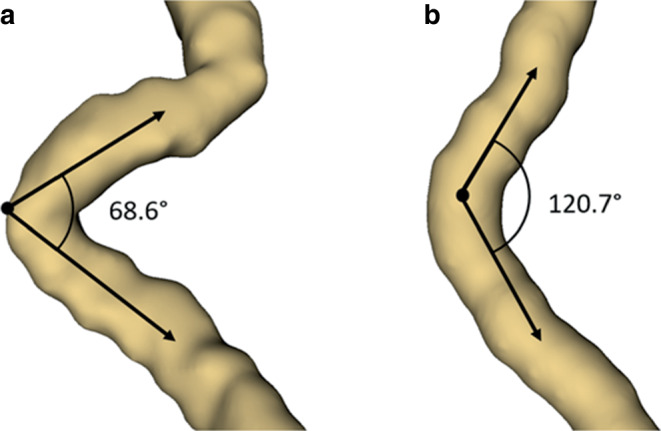
Measurement of two exemplary angles on segmented ICAs in 3D Slicer. **a** Shows an ICA angle of 68.6°, **b** shows an ICA angle of 120.7°

Figure 2 shows the measurement of two exemplary angles.

The continuous angles were explored regarding their distribution as well as correlation with procedural times. Furthermore, a cut-off analysis to find optimal thresholds for classification was performed. For this purpose, we divided all cases in “long” cases with groin puncture to first maneuver above the 80th percentile (41 min and longer) as well as groin puncture to final TICI above the 80th percentile (113 min and longer). Optimal thresholds of ICA angles for best discrimination between “long” cases versus all other cases were calculated using receiver operating characteristic curves by maximizing Youden’s index and running 1000 bootstrap repeats. The performance of the thresholds was assessed using a machine-learning classification algorithm. On basis of these analyses, ICAs were grouped into two classes (acute angle ≤ 90° or obtuse angle > 90°). This dichotomization was performed to enable classification with a neural network as well as to ensure comparability of results with previous works [18]. Of the ICAs 66 were not assessable as they were not continuously contrasted in the CTA (either due to late contrast phases or proximal higher grade stenoses that were not treated in the acute setting). After class formation, the association between the manually measured ICA angle on the occluded side in relation to EVT duration, as well as the interval from groin puncture to first thrombectomy maneuver was explored.

### ICA Angle Classification with CNN

To facilitate the following DL-based classification task, a preprocessing was designed, consisting of the following steps:

1. Cropping of the volume to the minimum extent, which includes the segmentation.
2. Creating two volumes, one containing the right ICA, and one containing the left ICA.
3. Changing the spacing to 0.5 mm.
4. Extending the volume size to 400 × 400 × 605 by padding
5. Rescaling to a size of 128 × 128 × 196

**Fig. 3:**
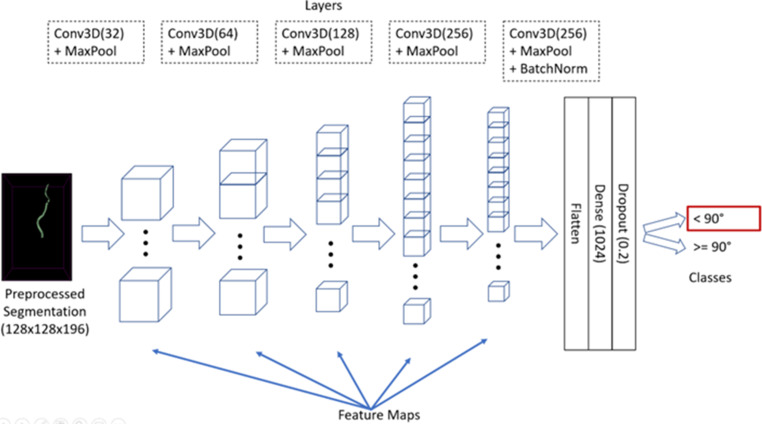
Network set-up for the ICA angle classification task detailing the input, layers used as well as output format. The dotted rectangles contain information about the respective layer composition. Conv3D: Convolutional Layer with number of filters in parenthesis; MaxPool: Layer to reduce overfitting and reduce computational cost; BatchNorm: facilitates faster training as it enables higher learning rates

A 3D CNN was designed according to Zunair et al. and Ertl et al. [28, 29]. Figure 3 gives an overview of the general network setup. A *Rectified Linear Unit* was used as the activation function except for the last layer, which has a sigmoid activation function. *Binary Cross Entropy* was used as the loss function and *Adam* as the optimizer. The training was performed over 100 epochs with a learning rate of 0.0001 and a batch size of 2. In terms of data augmentation, rotation in two planes was applied stochastically and with random angles and mirroring. The input for the network was the segmentation of a CCA + ICA. The output of the network was 0 or 1 with 0 relating to the class ICA angle 90° and 1 to the class ICA angle ≤ 90°. The network was implemented with keras and tensorflow (DL frameworks for the python programming language).

The training set specified after data export was further split into five folds for cross-validation. Five models were trained, each time using a different fold as the validation set and the other four as the training set. Training was performed on a machine running Ubuntu 20.04 (Canonical, London, United Kingdom) with 6 cores, 64 GB RAM and an Nvidia (Nvidia, Santa Clara, CA, USA) Titan RTX.

Each of the five trained models predicted on the test set and their predictions were averaged. Performance was assessed by accuracy and AUC.

The ICA angle predictions of the model on the test set were then again explored regarding association in relation to EVT duration as well as the interval from groin puncture to first maneuver.

### Statistical Analysis

Statistical analysis was performed using the scipy library in python as well as R version 4.0.0 (Foundation for Statistical Computing, Vienna, Austria). Missing values for EVT duration and groin puncture to first maneuver were imputed with median (2 and 6 values, respectively). Testing for normality was performed using Shapiro-Wilk and testing for statistical significance using Wilcoxon-Mann-Whitney‑U, accordingly. Pearson’s rho was calculated for correlation analyses. A *p*-value < 0.05 was considered significant.

## Results

A total of 379 patients were included for the development of DL models. After exclusion of tandem occlusions 348 patients were used for analysis regarding procedural times. An overview of clinical and procedural data of these patients is shown in Table 2. Patients presented with a median NIHSS at baseline of 15 (interquartile range, IQR 10–20) and median ASPECTS of 8 (IQR 7–9). Median onset to recanalization time was 353 min (IQR 242–473min) with 57% achieving successful recanalization (defined as mTICI ≥ 2c). Good clinical outcome (mRS 0–2 after 90 days) was attained in 33% of patients.

**Table 2:** Overview of clinical and procedural parameters of the patient collective

Parameters	Patients (*n* = 348)
Age (years): mean (± SD)	74.9 (± 12.1)
Right side occluded: *n* (%)	186 (53%)
Baseline NIHSS: median (IQR)	15 (10–20)
NIHSS 24 h: median (IQR)	10 (4–19)
Baseline ASPECTS: median (IQR)	8 (7–9)
Onset to groin puncture (min): median (IQR)	270 (180–473)
Onset to recanalization (min): median (IQR)	353 (242–557)
Groin puncture to first maneuver (min): median (IQR)	26 (19–38)
EVT duration (min): median (IQR)	58 (34–103)
Fluoroscopy time (min): median (IQR)	33 (17–59)
Stent-retriever maneuvers: mean (±SD)	1.9 (± 2.0)
Aspiration maneuvers: mean (±SD)	0.7 (± 1.0)
Only aspiration: *n* (%)	145 (42%)
IV rtPA: *n* (%)	151 (43%)
HBC 1c: *n* (%)	22 (6%)
HBC 2: *n* (%)	12 (3%)
*Prestroke mRS: n (%)*	
0	119 (34%)
1	80 (23%)
2	55 (16%)
3	67 (19%)
4	21 (6%)
5	6 (2%)
*mRS-90: n (%)*	
0	29 (8%)
1	38 (11%)
2	48 (14%)
3	63 (18%)
4	54 (16%)
5	32 (9%)
6	84 (24%)
*TICI score post intervention: n (%)*	
≤ 2b	148 (43%)
2c/3	199 (57%)

*NIHSS* National Institutes of Health Stroke Scale, *ASPECTS* Alberta Stroke Program Early CT score, *rtPA* recombinant tissue-type plasminogen activator, *HBC* Heidelberg bleeding classification, *mRS* modified Rankin Scale, *TICI* thrombolysis in cerebral infarction, *SD* standard deviation, *IQR* interquartile range

### Vessel Tree Segmentation

**Fig. 4:**
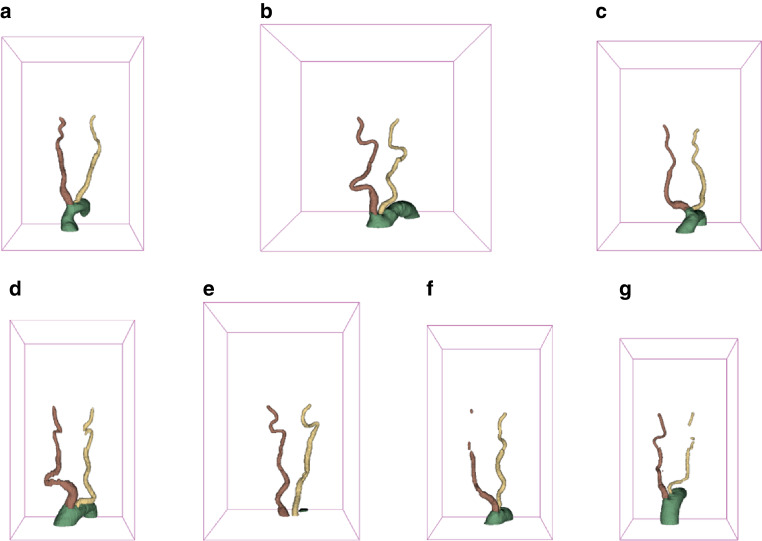
Exemplary segmentations of cases with special characteristics. All segmentations shown are predictions of the trained nnUNet on the test set. **a** Segmentation of a vessel tree with limited tortuosity. **b** Vessel tree with extensive tortuosity. **c** Vessel tree with bovine arch configuration. **d** Vessel tree from low-resolution CT (Y-axis spacing = 3 mm). **e** Vessel tree from CT that did not include aortic arch in the raw data. **f** Vessel tree from CT with right ICA not filled with contrast agent due to distal ICA occlusion. **g** Vessel tree with parts of left jugular vein mistaken for left ICA

Evaluation against the test set of the trained nnUNet resulted in Dice scores of 0.944, 0.854, and 0.856 for the aorta, left CCA/ICA, and right CCA/ICA, respectively, summarized in Table 3. Table 4 shows the time efforts for manual and hybrid segmentation. Prediction of a segmentation took 30 s with another 3 min on average for manual verification. The hybrid segmentation was 11 times faster than manual segmentation. Figure 4 gives an overview of the segmentations resulting from the trained nnUNet.

**Table 3:** Evaluation metrics of the trained nnUNet against the test set

Metric/structure	Aorta	Left CCA/ICA	Right CCA/ICA
Dice	0.944	0.854	0.856
precision	0.943	0.826	0.863
recall	0.945	0.893	0.871

*CCA* common carotis artery, *ICA* internal carotid artery

**Table 4:** Time efforts for manual and hybrid segmentation

Approach	*N* of CTA segmented	Time total (h)	Time per CTA (h)
Manual	121	80	Mean: 0.66, median: 0.70
Hybrid	258	15	0.06

*CTA* Computed tomography angiography

### Analysis On Continuous ICA Angles

**Fig. 5:**
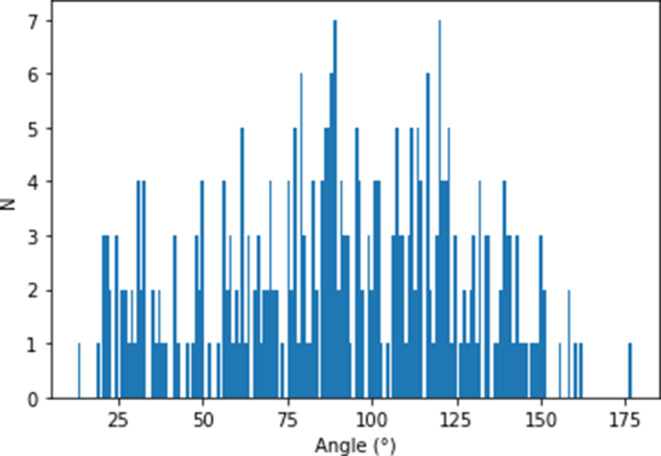
Quantitative distribution of ICA angles measured on the occluded side in a histogram

Figure 5 shows the distribution of ICA angles on the occluded side and Table 5 gives an overview about descriptive measures regarding the manually measured angles on the occluded side. The correlation analysis between ICA angles and procedural time showed a significant but weakly negative correlation for EVT duration (Pearson’s r = −0.12, *p* < 0.022), while for the correlation between ICA angles and time to first maneuver, no significant correlation was found (Pearson’s r = −0.09, *p* = 0.097). For groin puncture to first maneuver, the optimal threshold was found at 89° (accuracy 0.61) and for groin puncture to final TICI was found at 92° (accuracy 0.55). Based on these findings, the best overall discriminatory power was derived for an angle of 90°, which was further used for classification.

**Table 5:** Descriptive statistics regarding the manually measured angles on the occluded side

*N*	316
Mean (°)	91.0
Median (°)	91.5
Standard deviation (°)	35.9
Interquartile range (°)	38.0–129.5

### Association Between Dichotomized ICA Angles and Procedural Times

Of the 758 ICAs assessed, 357 ICAs were classified manually as having an acute inner angle of ≤ 90° and 335 as having an obtuse inner angle of > 90°, while 66 were not assessable, of which 32 were on the occluded side. After exclusion of the cases with tandem occlusions, 316 occluded side ICAs were tested for association with procedural times.

**Fig. 6:**
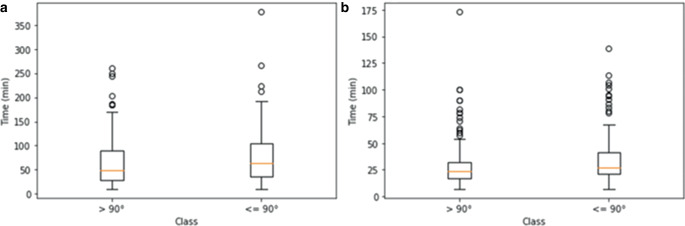
Box-and-whisker plots depicting the relationship between manually measured ICA angle and **a** EVT duration and **b** groin puncture to first maneuver

Testing for normal distribution was negative, therefore the Wilcoxon-Mann-Whitney test was used to explore statistical significance. The angle of the ICA ipsilateral to the occlusion was strongly associated with the overall procedure time (i.e. groin puncture to first thrombectomy maneuver) with a *p* = 0.001. The angle of the ICA ipsilateral to the occlusion was strongly associated with the EVT duration as well as the interval from groin puncture to first maneuver, both with a *p* = 0.001. Table 6 and Fig. 6 summarize the results of the ICA angle classification.

**Table 6:** Descriptive statistics regarding the association between internal carotid artery (ICA) angle and endovascular thrombectomy (EVT) duration/groin puncture to first maneuver

ICA angle	≤ 90°	> 90°
*EVT duration*		
*N*	162	154
Median (°)	48.0	63.5
Mean (°)	64.4	80.6
Standard deviation (°)	50.3	57.0
Interquartile range (°)	61.0	68.25
*Groin puncture to first maneuver*		
*N*	162	154
Median (°)	23.5	27.5
Mean (°)	29.1	34.3
Standard deviation (°)	21.3	22.4
Interquartile range (°)	14.75	20.0

*EVT* Endovascular thrombectomy

### ICA Angle Classification with CNN

Both left and right ICAs of the 379 patients were used for the angle classification task. As 66 ICAs were not assessable, this resulted in 587 for the train set and 105 ICAs for the test set. Table 7 gives an overview of the train/test distribution.

**Table 7:** Class and split distribution

Set/class	> 90°	≤ 90°	Total
Train (*n*)	304	283	587
Test (*n*)	53	52	105
Total (*n*)	357	335	692

**Fig. 7:**
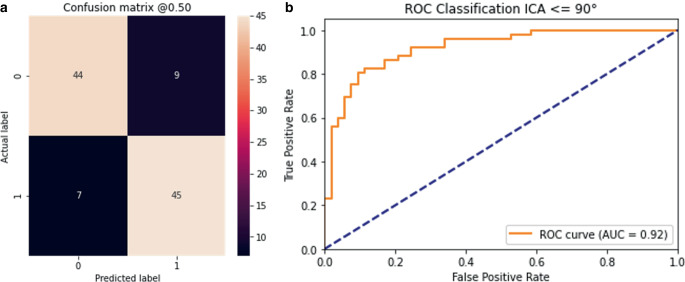
Confusion matrix (**a**) and ROC curve (**b**) evaluating the classification network against the test set during the ICA classification task. Label 0 in the confusion matrix represents the class ICA angle > 90° and label 1 ICA angle ≤ 90°

Prediction of the 105 test ICAs by the trained networks took 18 s, 0.2 s per ICA. The evaluation against the test set of the trained CNN resulted in an AUC of 0.92 and an accuracy of 0.85. Figure 7 shows the evaluation against the test set for the ICA angle classification task. The online only supplemental Figure S1 shows the training progress of the fivefold cross-validation.

**Fig. 8:**
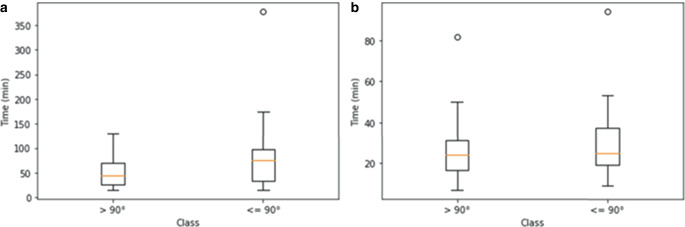
Box-and-whisker plot depicting the relation between ICA angle and **a** EVT duration as well as **b** groin puncture to first maneuver as by the predictions of the trained CNN on the test set

Of the test ICAs 49 were on the occluded side with 2 of them being tandem occlusions, resulting in 47 ICAs that were evaluated for procedural times. Testing for significance regarding the predicted ICA angles on the test set against EVT duration (*p* = 0.10) and groin puncture to first maneuver (*p* = 0.29) was negative although descriptive statistics revealed differences, especially for EVT duration (median 44.0 min for ICA angle > 90° and 75.5 min for ICA angle ≤ 90°, see Table 8; Fig. 8).

**Table 8:** Descriptive statistics regarding the association between internal carotid artery (ICA) angle and endovascular thrombectomy (EVT) duration/groin puncture to first maneuver as by the predictions of the trained CNN on the test set

ICA angle	> 90°	≤ 90°
*EVT duration*		
*N*	23	24
Median (°)	44.0	75.5
Mean (°)	55.0	85.8
Standard deviation (°)	34.9	73.6
Interquartile range (°)	43.5	63.75
*Groin puncture to first maneuver*		
*N*	23	24
Median (°)	24.0	25.0
Mean (°)	27.0	30.3
Standard deviation (°)	15.6	18.1
Interquartile range (°)	14.5	17.75

*EVT* Endovascular thrombectomy

## Discussion

The duration of EVT is an important predictor for neurological outcome after endovascular stroke treatment [2], and the anatomy of the extracranial vasculature was shown to significantly affect EVT duration [11–15, 17]. To rapidly predict difficulties in the vascular access we aimed at developing tools for automatic assessment of extracranial vasculature.

In this work, a robust approach to semi-automatically detect difficulties in the cervical vascular access in patients with acute ischemic stroke with our CNN showing very high performance in identifying critical ICA angles could be demonstrated. Furthermore, we were able to verify the reported association between acute ICA angle ≤ 90° and EVT duration by Holswilder et al. [18] using manual measurements that were used as a ground truth for our CNN approach. Additionally, we showed that this association is given as well for groin puncture to first maneuver which is in accordance with Snelling et al., Rosa et al. and Mokin et al. [12, 13, 17]; however, when testing with the predictions of the trained CNN, no statistical significance between the groups was detected. This might be due to the limited number of cases, as only the cases from the test set could be used to assess the network’s predictions.

Work regarding UNet-based segmentation of neck vessels had already been published [22, 23]. We differ from the reported approaches in that we gave distinct labels to distinct parts of the vessel tree. This facilitates automatic selection of the vessel of interest. Furthermore, we included CTAs of a Y-axis spacing up to 3 mm in our work to provide robustness of network performance across CTAs of varying resolution. Also, the performance metrics reported are similar to ours with Fu et al. reporting a Dice score of 0.94 and Wang et al. of 0.91, while we achieved 0.94 and 0.85, respectively, for our different labels.

To assure segmentation integrity for the classification task, we decided to verify DL-generated segmentations. For clinical use, this step should be eliminated. Manual verification might be rendered unnecessary by mobilizing a larger dataset for training. As the segmentation part is the only step in our approach that requires manual intervention, we assume that our vessel assessment holds the potential to become fully automatic.

Approaches for semi-automatic assessment of extracranial vasculature for EVT has been published by Mokin et al. [17]. Their method also relies on segmentation with vessel centerline determination afterwards. While vessel centerline assessment is less prone to interobserver variability than angle measurement, the required time to assess a patient of 15–20 min limits its clinical use.

Existing work has already proposed scores to grade extracranial vasculature for difficulty regarding EVT, namely the B.A.D. [12] and ASMETS [13] scores. These scores rely on the aortic arch, CCA, and ICA morphology. As our DL-based segmentation approach already includes these structures, our work for classifying ICA angles can be extended easily to facilitate (semi‑)automatic collection of these scores. This holds the potential to ease the load on physicians in the time-critical context of performing EVT. Another field with high clinical importance is the prediction of failed femoral access in patients undergoing EVT [30]. We are planning further work to predict conversion from femoral to radial access from further vessel features such as the aortic arch configuration and believe that this study provides a solid base to achieve this task and to provide tools for objective patient assessment in the clinical context.

There are limitations in this study that need to be acknowledged. Foremost, a limitation of both the presented classification and the segmentation task is that they represent the results of a retrospective monocentric study and were not yet verified on external data, which will be the target of future studies. The emerging practice of federated learning can be discussed to accomplish performance improvement and generalizability over multicentric data at once [31, 32]. Furthermore, for this analysis only M1 occlusions were used for development. Additionally, the angle measurement was performed in a plane on three-dimensional segmentations but was not a true three-dimensional angle. Also, we must need to acknowledge the limitations of dichotomization of quantitative data, that we undertook by classifying the angle measurements and that can possibly lead to a substantial information loss that might have impacted the results. Moreover, some of the ICA segmentations were not assessable due to insufficient contrast filling either due to pseudo-occlusions, higher grade proximal ICA stenoses or technical issues, which might have created a bias in our results.

In conclusion, we could demonstrate a combined DL-based segmentation and classification approach for the assessment of difficult cervical vascular anatomy in patients with acute ischemic stroke. The potential for full automation exists and could be used for fast and reliable triage and enhanced allocation of material and staff to further improve the clinical outcome of stroke patients. With our work, we would also like to pave the way for answering additional important questions, such as the failure of a femoral access or the choice of guiding catheters needed to overcome difficult vascular anatomy.

## Supplementary Information

Figure S1: Training progresses of the 5 models generated during cross-validation for the network trained for ICA angle classification

## Abbreviations

AIS: Acute ischemic stroke
ASPECTS: Alberta Stroke Program Early CT score
AUC: Area under the curve
CCA: Common carotid artery
CNN: Convolutional neural network
CTA: Computed tomography angiography
DL: Deep learning
EVT: Endovascular thrombectomy
HBC: Heidelberg bleeding classification
ICA: Internal carotid artery
LVO: Large vessel occlusion
MRS: Modified Rankin Scale
NIHSS: National Institutes of Health Stroke Scale
RtPA: Recombinant tissue-type plasminogen activator
TICI: Thrombolysis in cerebral infarction
