# Supplementary material for: Deep Learning-based Assessment of Internal Carotid Artery Anatomy to Predict Difficult Intracranial Access in Endovascular Recanalization of Acute Ischemic Stroke
Source: Clin Neuroradiol. 2023 Mar 16;33(3):783–92. doi: 10.1007/s00062-023-01276-0 (PMC10449951; doi:10.1007/s00062-023-01276-0)
Supplement: Supplementary file 1 — Figure S1: Training progresses of the 5 models generated during cross-validation for the network trained for ICA angle classification [file 62_2023_1276_MOESM1_ESM.pdf]

SUPPLEMENTAL MATERIAL

Supplemental Figures

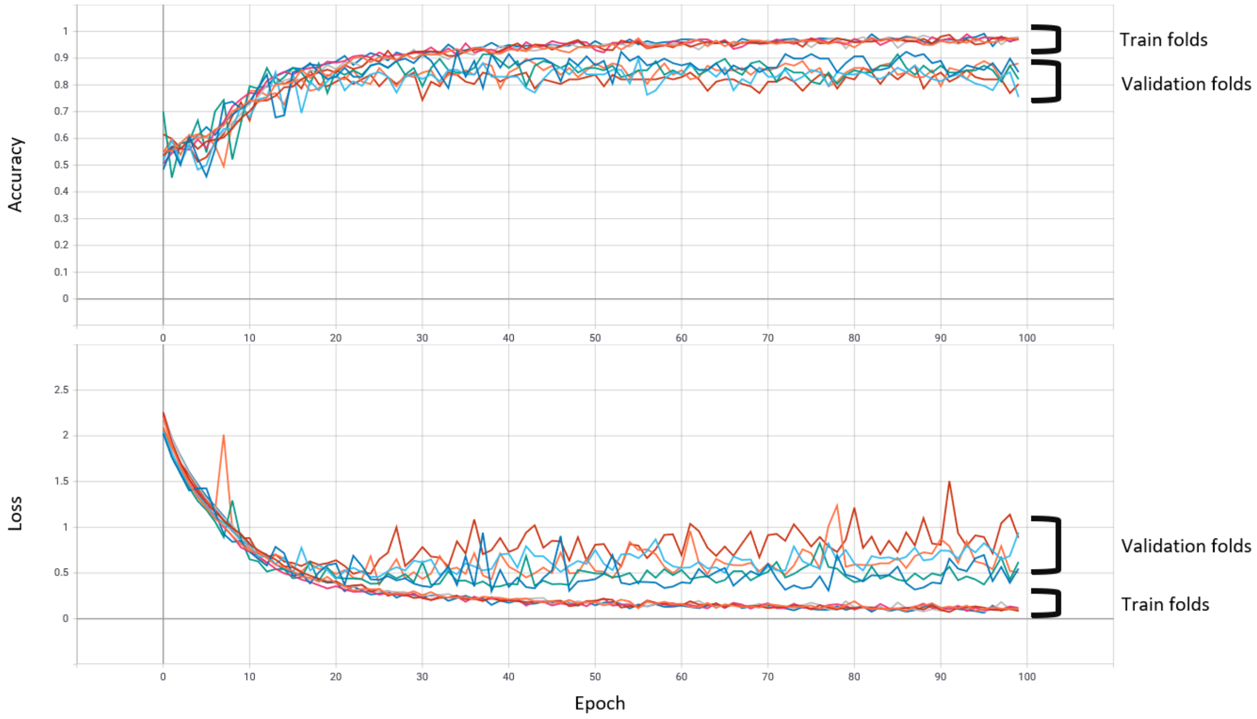

Figure S1: Training progresses of the 5 models generated during cross-validation.
